# IFNɣ but not IFNα increases recognition of insulin defective ribosomal product-derived antigen to amplify islet autoimmunity

**DOI:** 10.1007/s00125-023-05991-8

**Published:** 2023-08-15

**Authors:** Sofia Thomaidou, Amadeo Munoz Garcia, Sabine de Lange, Jin Gan, Arno R. van der Slik, Rob C. Hoeben, Bart O. Roep, Françoise Carlotti, Arnaud Zaldumbide

**Affiliations:** 1 Department of Cell and Chemical Biology, Leiden University Medical Center, Leiden, the Netherlands; 2 Department of Internal Medicine, Leiden University Medical Center, Leiden, the Netherlands

**Keywords:** Autoantigens, Degradation, Inflammation, Proteasome, Type 1 diabetes

## Abstract

**Aims/hypothesis:**

The inflammatory milieu characteristic of insulitis affects translation fidelity and generates defective ribosomal products (DRiPs) that participate in autoimmune beta cell destruction in type 1 diabetes. Here, we studied the role of early innate cytokines (IFNα) and late immune adaptive events (IFNɣ) in insulin DRiP-derived peptide presentation to diabetogenic CD8+ T cells.

**Methods:**

Single-cell transcriptomics of human pancreatic islets was used to study the composition of the (immuno)proteasome. Specific inhibition of the immunoproteasome catalytic subunits was achieved using siRNA, and antigenic peptide presentation at the cell surface of the human beta cell line EndoC-βH1 was monitored using peptide-specific CD8 T cells.

**Results:**

We found that IFNγ induces the expression of the *PSMB10* transcript encoding the β2i catalytic subunit of the immunoproteasome in endocrine beta cells, revealing a critical role in insulin DRiP-derived peptide presentation to T cells. Moreover, we showed that *PSMB10* is upregulated in a beta cell subset that is preferentially destroyed in the pancreases of individuals with type 1 diabetes.

**Conclusions/interpretation:**

Our data highlight the role of the degradation machinery in beta cell immunogenicity and emphasise the need for evaluation of targeted immunoproteasome inhibitors to limit beta cell destruction in type 1 diabetes.

**Data availability:**

The single-cell RNA-seq dataset is available from the Gene Expression Omnibus (GEO) using the accession number GSE218316 (https://www.ncbi.nlm.nih.gov/geo/query/acc.cgi?acc=GSE218316).

**Graphical Abstract:**

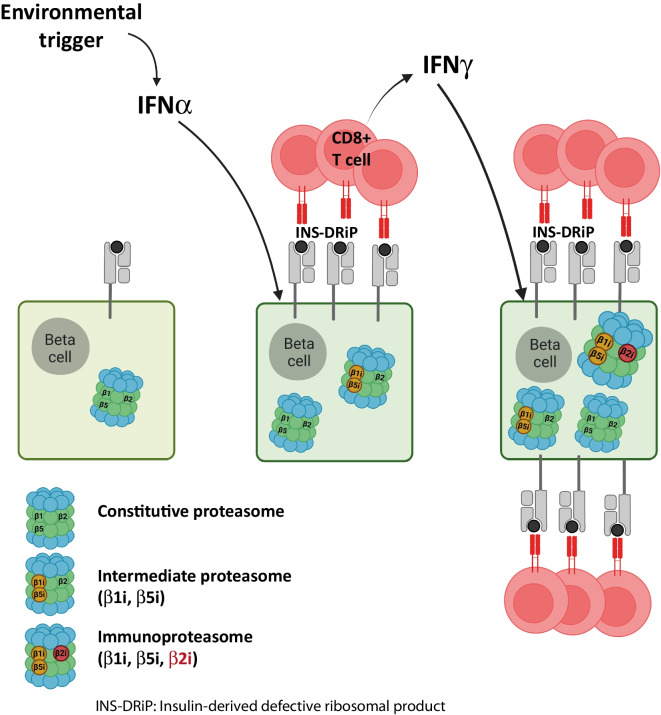

**Supplementary Information:**

The online version of this article (10.1007/s00125-023-05991-8) contains peer-reviewed but unedited supplementary material.


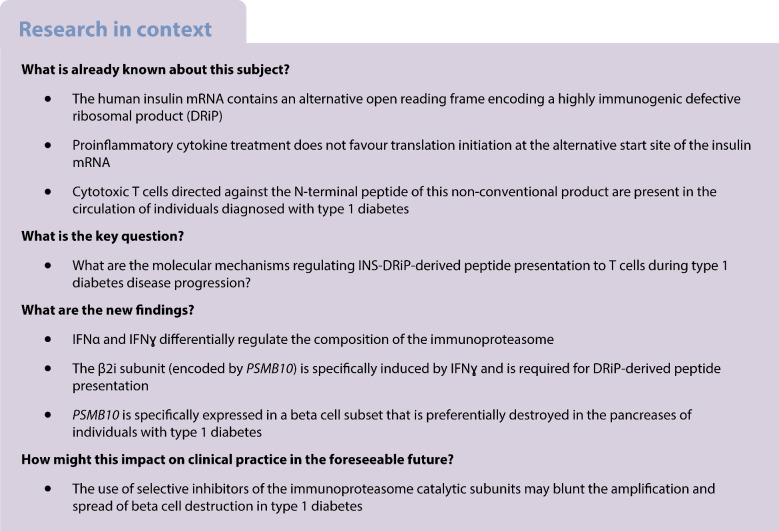


## Introduction

Type 1 diabetes is characterised by the progressive destruction of insulin-producing beta cells by CD8+ T cells [1]. While impaired central and peripheral immunological tolerance in combination with low-affinity T cells have been suspected to play a role in the immune attack [2], the demonstration that naive autoreactive T cells are part of the normal T cell repertoire supports the participation of beta cells and the islet microenvironment in triggering or driving disease progression [3, 4]. We have shown that local inflammation and the unfolded protein response to stress disturb the cellular equilibrium and affect translation fidelity, generating defective ribosomal products (DRiPs) and alternative reading frame-encoded peptides that the immune system is trained to act on [5]. Although the molecular mechanisms leading to processing and presentation of these ‘junk’ polypeptides remain unclear, the presence of insulin-derived defective ribosomal product (INS-DRiP)-specific CD8 T cells, with an effector phenotype in donors with type 1 diabetes, supports their potential relevance as autoimmune T cell targets [6, 7].

Upon inflammation or during cellular stress, misfolded proteins that have accumulated in the ER are redirected to the ubiquitin–proteasome system pathway for degradation to restore cellular homeostasis. Dysregulation of this system and inappropriate processing may lead to cytoplasmic aggregates and the manifestation of neurodegenerative diseases [8] and type 2 diabetes [9], as well as to the development of autoimmunity. In proteasome-associated autoinflammatory syndromes, mutations within the genes encoding the immunoproteasome catalytic subunits are associated with an excessive IFN response [10]. The proteasome is the main component of the antigen presentation machinery. In homeostatic conditions the proteasome consists of a 20S catalytic barrel and two regulatory 19S complexes, forming the 26S holo structure. Within the 20S core reside the β subunits—β1 (encoded by *PSMB6*), β2 (*PSMB7*) and β5 (*PSMB5*)—which display caspase, trypsin and chymotrypsin cleavage specificities, respectively. Upon inflammation, these subunits are partially or completely substituted by the induced counter forms β1i/low-molecular mass polypeptide (LMP)2 (encoded by *PSMB9*), β2i/multicatalytic endopeptidase complex-like-1 (MECL1; *PSMB10*) and β5i/LMP7 (*PSMB8*), respectively, to generate intermediate or full immunoproteasomes [11], affecting proteolytic activity and producing qualitative or quantitative differences in the peptide ligandome presented by MHC class I molecules [12, 13].

In this study, we investigated the composition of the (immuno)proteasome in human beta cells under inflammatory conditions using single-cell RNA-seq analysis to determine the molecular mechanisms underlying the immunogenicity of INS-DRiP.

## Methods

### Cells and reagents

The human beta cell line EndoC-βH1 (Human Cell Design, Paris, France), mycoplasma free, kindly provided by R. Scharfmann (Paris Descartes University, France) [14], was maintained in low-glucose DMEM supplemented with 5.5 μg/ml human transferrin, 10 mmol/l nicotinamide, 6.7 ng/ml selenite, 50 μmol/l β-mercaptoethanol, 2% human albumin (wt/vol), 100 units/ml penicillin and 100 μg/ml streptomycin. Cells were seeded in extracellular matrix (fibronectin) pre-coated culture plates. Inflammatory stress was induced with IFNα (PBL Bioscience, USA), IFNγ (Bio-Techne, USA) or IL1β (Sigma-Aldrich, USA) at the concentrations and for the times indicated. INS-DRiP-directed cytotoxic T lymphocytes (CTLs) were isolated from freshly isolated peripheral blood mononuclear cells (PBMC) from a long-term HLA-A2^+^ individual with type 1 diabetes. As described previously [7], 150,000 PBMC/well were seeded with 10 μg/ml DRiP_1–9_ peptide in Iscove's modified Dulbecco's medium (IMDM; Life Technologies, USA) supplemented with 10% human serum, 0.5% LeucoA, 0.1 ng/ml IL-12, 10 ng/ml IL-7, 25 U/ml IL-2 and 5 ng/ml IL-15. After 14 days of culture, cells were restimulated specifically with irradiated DRiP_1–9_ peptide-pulsed JY cells (2 μg/ml peptide with 10 × 10^6^ cells in AIM-V medium (Life Technologies, USA) for 2 h at 37°C and 100,000 cells/well irradiated allogeneic PBMCs in IMDM supplemented with human serum and cytokines as described above. JY (ATCC 77441) cells, mycoplasma free, were maintained in IMDM supplemented with 8% FCS, 100 U/ml penicillin and 100 μg/ml streptomycin.

### Lentivirus production and transduction

The lentiviral vector containing HLA-A*02:01 under the elongation factor 1α (EF1α) promotor was obtained from R. J. Lebbink (Medical Microbiology, University Medical Center Utrecht, Utrecht, the Netherlands) [16] and produced as described previously [15]. Briefly, the EF1α-HLA-A*02:01 containing lentiviral vector and the three ‘helper’ plasmids (encoding HIV-1 gag–pol, HIV-1 rev and VSV-G envelope) were co-transfected overnight using polyethylenimine into 293T cells. The medium was refreshed and viruses were harvested after 48 and 72 h, passed through 0.45 μm filters and stored at −80°C. Viral supernatants (multiplicity of infection [MOI]=2) were added to EndoC-βH1 cells in fresh medium supplemented with 8 μg/ml Polybrene (Sigma-Aldrich, USA) and the cells were incubated overnight.

### siRNA transfection

Transfection of siRNAs (SMARTpool) was performed using the Dharmacon transfection reagent (Dharmafect1) according to the manufacturer’s instructions (Horizon Discovery, USA). EndoC-βH1 cells were transfected in 12-well plates for 72 h, using a final concentration of 5 nmol/l total siRNA pool mix.

### RT-PCR

Total RNA was extracted from EndoC-βH1 cells using the NucleoSpin Kit (740609.50S, Bioke, the Netherlands). Approximately 0.5 µg of RNA was used for reverse transcription. Oligo (dT) primers were used in the reactions. Expression of the transcript of interest was detected using primers listed in ESM Table 1.

### Western blot analyses

EndoC-βH1 cells were lysed in RIPA buffer supplemented with a protease inhibitor cocktail (Roche Applied Science, Germany). Protein quantification was performed using the BCA protein assay kit (Thermo Fisher Scientific, USA). A total of 25 µg of protein was subjected to electrophoresis on 12% acrylamide/bis acrylamide SDS page gels. After electrophoresis, proteins were transferred onto nitrocellulose membranes (GE Healthcare, USA). Membranes were stained with primary antibodies overnight at 4°C and secondary HRP-conjugated antibodies (Santa Cruz Biotechnology, USA) for 1h at room temperature. Primary antibodies were from Enzo Life Sciences (Switzerland) and were used at a dilution of 1:1000 (β1i: BML-PW8345; β5i: BML-PW8355; β2i: BML-PW8350). The loading control was β-actin (MAB1501, EMD Millipore, USA) and was used at 1:1000 dilution. Secondary antibodies were anti-mouse (#G21040) or anti-rabbit (#sc-2004) antibodies from Santa Cruz Biotechnology and were used at a dilution of 1:5000. Western ECL substrate was used for imaging (1705062, BioRad, USA).

### Pancreatic islet treatment

Pancreatic islets used in the T cell co-culture assays were obtained from a human organ donor. Research consent was obtained according to national law and regulations. The islets were isolated in the GMP (good manufacturing practice) facility of Leiden University Medical Center according to a previously reported protocol [17]. For experimental use, human islets were maintained in ultra-low attachment plates (Corning, USA) in low-glucose DMEM supplemented with 10% FBS, 100 U/ml penicillin and 100 μg/ml streptomycin. Dispersed islet cells were treated with 1000 U/ml IFNγ or 2000 U/ml IFNα for 24 h. All methods were performed in accordance with relevant guidelines and regulations.

Human islets used in single-cell RNA-seq were provided through the Integrated Islet Distribution Program (IIDP). Pancreatic islets, isolated from three healthy donors, with a purity of at least 90%, were cultured on receipt in regular CMRL 1066 medium (5.5 mmol/l glucose) supplemented with 10% FCS, 20 mg/ml ciprofloxacin, 50 mg/ml gentamycin, 2 mmol/l l-glutamine, 10 mmol/l HEPES and 1.2 mg/ml nicotinamide. Islets were maintained in culture at 37°C in a 5% CO_2_ humidified atmosphere and medium was refreshed on receipt and every 2 days thereafter. Intact islets were treated with the following cytokines: 2000 U/ml IFNα and a combination of 1 ng/ml IL1β and 1000 U/ml IFNγ (R&D Systems, USA). After treatments, islets were dispersed into single cells using 0.025% trypsin (Gibco, USA) and 10 mg/ml Dnase (Pulmozyme, Genentech, USA). Single cells were then processed for single-cell RNA-seq following the standard 10x Genomics 3’ V3 chemistry protocol (10x Genomics, USA).

### RNA-seq data processing and analysis

Single-cell RNA-seq output was processed and analysed following the Seurat pipeline (version 4.0; https://cran.r-project.org/package=Seurat). Clustered cells were labelled and sorted into subsets based on the expression of canonical cell markers: insulin (beta cells), glucagon (alpha cells), somatostatin (delta cells), pancreatic polypeptide (pancreatic polypeptide/gamma cells), human cationic trypsinogen (acinar cells) and keratin 19 (duct cells). Differential gene expression analysis was performed using the Wilcoxon rank sum test to analyse gene expression differences between treated and untreated cell groups. The output of this analysis shows genes that are expressed in at least 25% of the cells in any group (treated or untreated). Bonferroni correction was applied to adjust the *p* values. Up-/downregulated genes (log fold change [FC] >0.5 and <–0.5 respectively) with an adjusted *p* value of <0.05 were considered to be significantly altered by the treatment.

Analyses of the pseudobulk differential expression of the endocrine cells was performed by clusterProfiler using the enrichPathway classification (https://bioconductor.org/packages/release/bioc/html/ReactomePA.html) [18].

### T cell activation assays

Target cells were harvested and co-cultured with CTLs specific for INS-DRiP_1–9_ (MLYQHLLPL) [7] or preproinsulin (PPI)_15–24_ (ALWGPDPAAA) [19] at an effector/target ratio of 2:1. Co-cultures were incubated at 37°C for 4 h in IMDM supplemented with 10% human albumin and 40 U/ml IL-2 (Novartis, Switzerland). The supernatant was used for detection of macrophage inflammatory protein-1 beta (MIP-1β) production by T cells, using the MIP-1β ELISA kit (88–7034–22, Thermo Fisher Scientific), according to the manufacturer’s protocol.

### Statistical analysis

Data are presented as means ± SEM. Calculations were performed using GraphPad Prism 7 (GraphPad Software, USA). Unpaired *t* tests were carried out for all comparisons.

## Results

### Type II but not type I IFN enhances INS-DRiP-derived peptide presentation to specific CTLs

Although we previously provided evidence for a role of INS-DRiP-directed T cells in beta cell destruction in type 1 diabetes [7], the contribution of the islet microenvironment that is characteristic of the early disease phase (the presence of type I IFN as a possible consequence of exposure to microorganisms, disturbed metabolism and tissue stress) or later disease phases (the presence of type II IFN resulting from activation of the adaptive immune response) remains unknown [20].

**Fig. 1:**
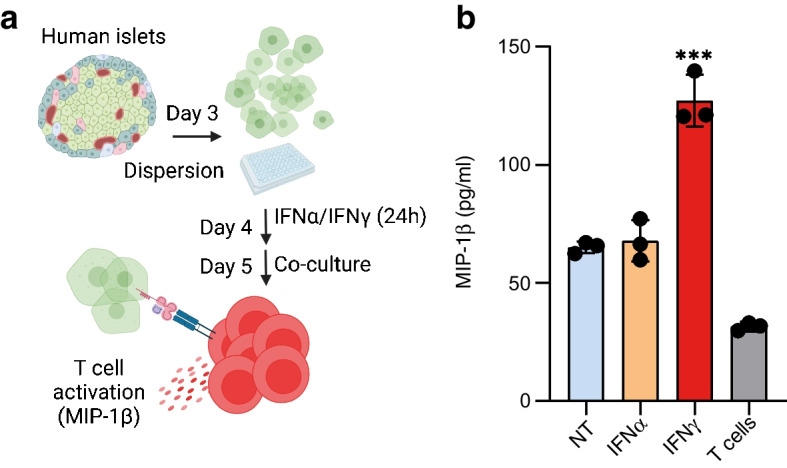
IFNα and IFNγ differentially regulate INS-DRiP presentation in human islets. (**a**) Schematic of the islet cells/INS-DRiP-specific CTLs co-culture experiment in the presence of 2000 U/ml IFNα or 1000 U/ml IFNγ. Created with BioRender.com. (**b**) MIP-1β secretion by INS-DRiP-specific CTLs after co-culture with HLA-A2^+^ primary human islet cells treated with IFNα or IFNγ for 24 h (*n*=3). NT, non-treated

To dissect whether and when INS-DRiP-derived peptide recognition is altered in the course of disease progression, we co-cultured dispersed HLA-A2+ human islets treated with IFNα (type I) or IFNɣ (type II) with an HLA-A2-specific CD8+ T cell clone, isolated from an individual with type 1 diabetes and directed against the N-terminal part of the INS-DRiP polypeptide (INS-DRiP_1–9_) [7] (Fig. 1a). Under these standardised conditions, previously described to mimic the islet inflammatory milieu [21–25], although IFNα did not trigger additional T cell activation compared with untreated islets, IFNɣ treatment caused an increase in MIP-1β secretion, indicating enhanced INS-DRiP peptide presentation (Fig. 1b). To validate the assay and confirm the correlation between the amount of peptide presented at the cell surface and the increase in MIP-1β secretion by T cells, we pulsed JY cells (HLA-A2+) with increasing titres of cognate peptide derived from INS-DRiP_1–9_ or HLA-A2 peptide derived from native PPI_15–24_. As expected, INS-DRiP CTLs were highly specific and the level of activation was proportional to the peptide concentration (ESM Fig. 1).

**Fig. 2:**
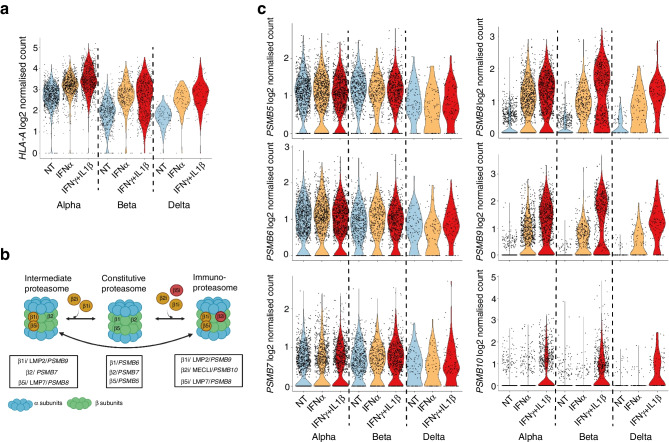
Cytokines differentially regulate immunoproteasome catalytic subunit expression in primary human endocrine cells. (**a**) Violin plots showing the expression levels of HLA-A after IFNα or IFNγ/IL1β treatment of endocrine cells. (**b**) Schematic representation of the composition of the proteasome and immunoproteasome. (**c**) Violin plots showing the expression levels of the mRNAs encoding the constitutive (*PSMB5*, *PSMB6* and *PSMB7*) and induced (*PSMB8*, *PSMB9* and *PSMB10*) catalytic subunits of the (immuno)proteasome. Expression levels correspond to log2 normalised counts/cell, as obtained in single-cell RNA-seq of human islets treated with IFNα (2000 U/ml) or IFNγ (1000 U/ml)+IL1β (2 ng/ml) for 24 h. NT, non-treated

Considering that HLA class I is the most important variable involved in antigen presentation to CD8 T cells, we used a single-cell RNA-seq dataset of human islets exposed to IFNα or IFNγ/IL1β (ESM Fig. 2) and evaluated the effect of both treatments on *HLA-A* expression in endocrine cells. As expected, both cytokines upregulated *HLA-A* expression to similar levels (Fig. 2a), suggesting that additional mechanisms are involved during protein processing after IFNɣ treatment to increase INS-DRiP-derived peptide presentation.

In our previous proteogenomic study [5], we demonstrated that IFNγ/IL1β treatment did not increase *INS* transcript expression nor favour ribosome docking to the DRiP start codon (as determined by long RNA-seq and ribosome profiling of EndoC-βH1 cells), suggesting that DRiP is produced equally in normal and inflamed conditions. Therefore, we reasoned that a difference in the protein degradation machinery may provide a rational explanation for the observed increase in INS-DRiP presentation after IFNɣ treatment. Using single-cell RNA-seq, we analysed the effect of IFNα and IFNγ/IL1β on the expression of the mRNAs encoding the constitutive catalytic subunits of the proteasome (β1 [encoded by *PSMB6*], β2 [*PSMB7*], β5 [*PSMB5*]) and the induced catalytic subunits of the immunoproteasome (β1i [encoded by *PSMB9*], β2i [*PSMB10*], β5i [*PSMB8*]) (Fig. 2b). While the constitutive subunits were not significantly affected by treatment, *PSMB8* and *PSMB9* mRNAs were upregulated by both IFNα and IFNɣ/IL1β in all endocrine and exocrine cell types (Fig. 2c and ESM Fig. 3). In contrast, the expression of *PSMB10* was significantly increased in endocrine alpha and beta cells after IFNɣ/IL1β treatment but not in delta or exocrine cells (ESM Table 2).

**Fig. 3:**
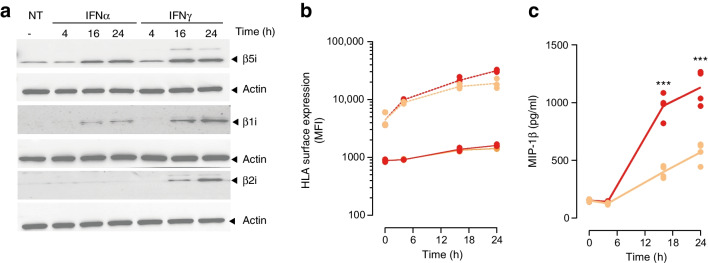
Proteasome composition in EndoC-βH1 cells after exposure to cytokines and sensitivity to INS-DRiP-specific CTLs. (**a**) β5i (encoded by *PSMB8*), β1i (*PSMB9*) and β2i (*PSMB10*) protein expression after incubation with 2000 U/ml IFNα or 1000 U/ml IFNγ determined by Western blot analysis. Actin was used as an internal control. Treatment duration was 4, 16 or 24 h, as indicated. (**b**) Surface expression of HLA-A/B/C (dashed lines) and HLA-A2 (solid lines) on EndoC-βΗ1-HLA-A2 cells treated with IFNα (orange lines) or IFNγ (red lines) for 4, 16 and 24 h. Values are presented as mean fluorescence intensity (MFI). (**c**) INS-DRiP-specific CTL activation determined by MIP-1β secretion after co-culture with EndoC-βΗ1-HLA-A2 cells treated with IFNα (orange line) or IFNγ (red line) for 4, 16 and 24 h. *n*=3 independent experiments. ***p≤ 0.001. NT, non-treated

To confirm these results and determine the main driver for the upregulation of *PSMB10* observed after IFNɣ/IL1β treatment in primary human beta cells, we exposed EndoC-βH1cells to recombinant IFNα, IFNɣ or IL1β for increasing amounts of time. As expected, we noted a rapid induction of β1i (encoded by *PSMB9*) and β5i (*PSMB8*) after IFNα and IFNɣ treatment and the absence of expression of β2i (*PSMB10*) in IFNα-treated cells (Fig. 3a). The absence of effect of IL1β on both HLA class I surface expression and immunoproteasome components (ESM Fig. 4) in these assays suggests that the expression of *PSMB10* observed in primary human islets on IFNɣ/IL1β treatment is mainly driven by type II IFN. To dissect the impact of cytokines on EndoC-βH1 cell immunogenicity, we generated a stable EndoC-βΗ1 cell line expressing HLA-A*02:01 by lentiviral transduction and exposed the cells for 4, 16 or 24 h to IFNα or IFNγ prior to co-culture with HLA-A2-restricted INS-DRiP-specific CD8+ T cells. We measured HLA class I expression in EndoC-βΗ1 cells and T cell activation by MIP-1β secretion. While IFNα and IFNγ differentially affected HLA class I surface expression, both treatments equally upregulated the expression of the HLA-A2 transgene (Fig. 3b). Under these conditions, and despite similar HLA-A2 expression levels, we observed an increase in T cell activation after IFNγ treatment, illustrating increased presentation of the DRiP-derived peptide to the specific CTLs, as observed for human islets (Fig. 3c).

### The β2i subunit enhances INS-DRiP presentation

**Fig. 4:**
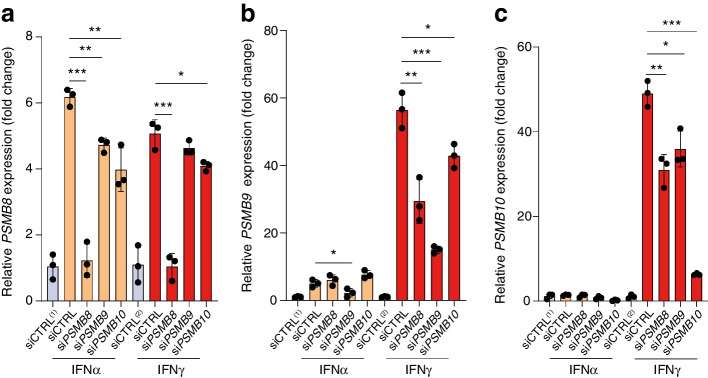
Immunoproteasome silencing in the EndoC-βΗ1 cell line. (**a**) *PSMB8*, (**b**) *PSMB9* and (**c**) *PSMB10* mRNA expression in EndoC-βΗ1-HLA-A2 cells transfected with non-targeted siRNA (siCTRL) and siRNAs specific for *PSMB8*, *PSMB9* and *PSMB10*. Cells were treated with 2000 U/ml IFNα (orange bars), 1000 U/ml IFNγ (red bars) or control medium (blue bars) 72 h post transfection for 24 h. Gene expression levels were corrected for levels of the housekeeping gene *GAPDH* and are presented as the induction ratio (relative to levels for siCTRL^(1)^ in IFNα samples and siCTRL^(2)^ for IFNγ samples) (*n*=3). *p≤ 0.05, **p≤ 0.01, ***p≤ 0.001

**Fig. 5:**
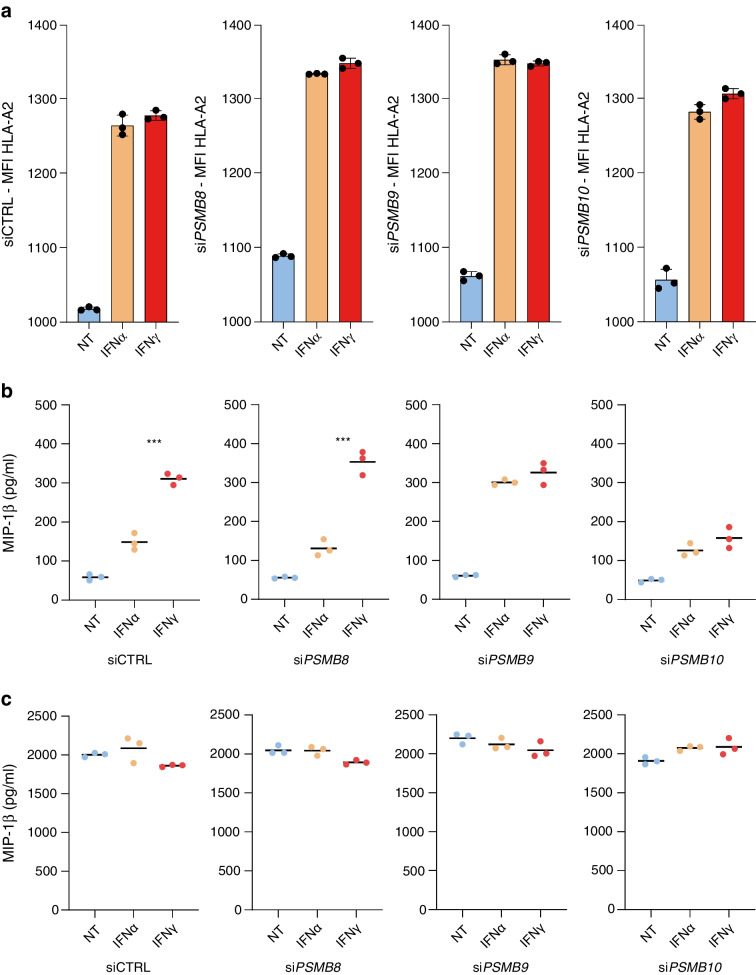
*PSMB10* silencing exclusively reduces INS-DRiP-specific CTL activation. (**a**) HLA-A2 (genetically introduced) surface expression of EndoC-βΗ1-HLA-A2 cells transfected with non-targeted siRNA (siCTRL) and siRNAs specific for *PSMB8*, *PSMB9* and *PSMB10*. Cells were left untreated (blue bars) or treated with 2000 U/ml IFNα (orange bars) or 1000 U/ml IFNγ (red bars) 72 h post transfection for 24 h. Values are presented as mean fluorescence (FITC) intensity (MFI) (*n*=3). (**b**, **c**) MIP-1β secretion by (**b**) INS-DRiP_1–9_-specific CTLs and (**c**) PPI_15–24_ after co-culture with EndoC-βΗ1-HLA-A2 cells. Prior to co-culture, target cells were transfected with non-targeted siRNA and siRNAs specific for *PSMB8*, *PSMB9* and *PSMB10*. Cells were left untreated (blue circles) or treated with IFNα (orange circles) or IFNγ (red circles) 72 h post transfection for 24 h (*n*=3). Statistical analyses are comparing IFNγ to IFNα treatments. ***p≤0.001. NT, non-treated

To determine the effect of the different immunoproteasome subunits on EndoC-βH1-HLA-A2 cell recognition by the INS-DRiP CD8+ T cell clone, siRNAs specific for *PSMB8*, *PSMB9* and *PSMB10* were used to selectively interfere with gene expression prior to IFNα and IFNγ treatment (Fig. 4a–c). We found that downregulation of the immunoproteasome catalytic subunits had no impact on HLA-A2 surface expression (Fig. 5a). To test for their immunogenicity, the modified beta cells were co-cultured with autoreactive T cells directed against INS-DRiP_1–9_ or PPI_15–24_ [26]. While activation of the PPI-specific T cell clone was unaltered after IFN treatment and immunoproteasome subunit modulation, *PSMB10* inhibition annihilated the IFNγ effect and reduced INS-DRiP-specific T cell reactivity to the levels seen after treatment with IFNα (Fig. 5b,c). Of note, the increased epitope presentation in the presence of IFNα after *PSMB9* knockdown is peculiar but may be related to a higher amount of intact epitope, as suggested by the presence in the epitope of hydrophobic residues targeted for chymotrypsin-like cleavage by β1i [27] (ESM Fig. 5). Accordingly, in silico analysis of the INS-DRiP sequence on the Proteasome Cleavage Prediction Server (PCPS) [28] shows that the INS-DRiP_1–9_ (MLYQHLLPL) 9-mer has a higher propensity to be processed by the immunoproteasome complex than by the constitutive proteasome. In addition, the localisation of trypsin digestion sites outside the 9-mer epitope region suggests that the β2i subunit may be involved in the processing of the INS-DRiP polypeptide but should not destroy the MLYQHLLPL epitope, meaning that its integrity is maintained.

### PSMB10, encoding the β2i subunit, is specifically expressed in a beta cell subset that is preferentially destroyed in pancreases of individuals with type 1 diabetes

**Fig. 6:**
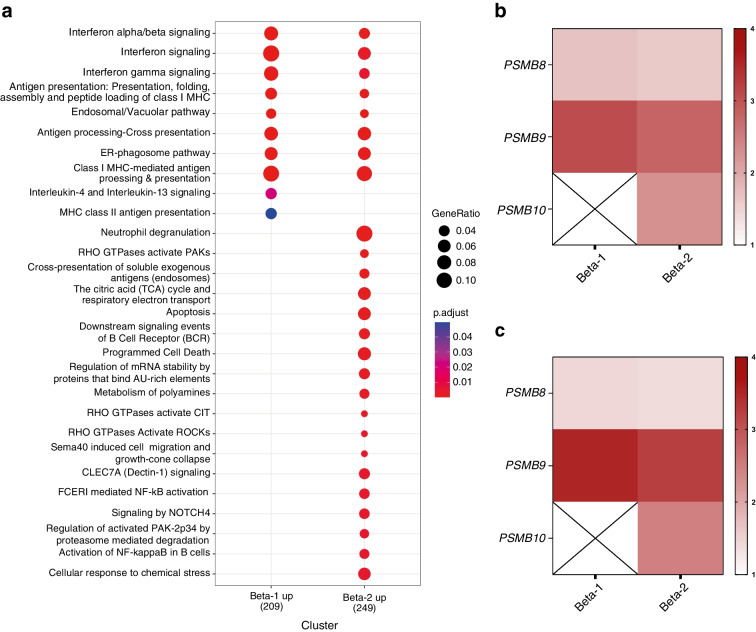
Immunoproteasome composition of beta cells from individuals with type 1 diabetes. (**a**) Gene ontology plot of transcripts upregulated in two distinct beta cell subsets (*p*<0.05 and logFC>1) in individuals with type 1 diabetes compared with control individuals (donors without diabetes). The plot was generated with clusterProfiler using the enrichPathway classification. (**b**) Heatmap showing the logFC (*p*<0.05) of *PSMB8*, *PSMB9* and *PSMB10* in the beta-1 and beta-2 populations from individuals with type 1 diabetes compared with control donors. (**c**) Heatmap showing the logFC (*p*<0.05) of *PSMB8*, *PSMB9* and *PSMB10* in the beta-1 and beta-2 populations from individuals with type 1 diabetes patients compared with AAb+ individuals. This figure has been generated using data described in Fasolino et al, 2022 [29]

We next assessed the potential clinical relevance of our findings in the context of the immunopathogenesis of type 1 diabetes using the single-cell multi-omics analysis launched by the Human Pancreas Analysis Program (HPAP) consortium [29]. This dataset identified two distinct beta cell clusters (beta-1 and beta-2) when comparing the transcriptomic profile of endocrine cells from donors with type 1 diabetes and endocrine cells from autoantibody-positive donors (AAb+). Those clusters are differentiated by the specific upregulation of apoptotic and adaptive immune system signalling in the beta-2 subset in AAb+, indicating that this cluster is undergoing cell death. Further examination of the pseudobulk differential expression analysis of the endocrine cells showed upregulation of the apoptosis pathway specifically in the beta-2 (‘minor’) beta cell subset (Fig. 6a, ESM Fig. 6 and ESM Table 3) and pointed to differences in the composition of the catalytic subunits of the immunoproteasome and to the selective expression of *PSMB10* in this subset (Fig. 6b,c). Altogether our data demonstrate the differential immune visibility of beta cells in the early and late phases of disease progression in type 1 diabetes and illustrate how IFNɣ can accelerate beta cell destruction by changing the composition of the immunoproteasome.

## Discussion

In this study we show that IFNα and IFNγ differentially regulate the proteasomal composition of beta cells and demonstrate that INS-DRiP peptide recognition may be involved in later phases of type 1 diabetes pathogenesis as an amplificatory phenomenon of beta cell destruction. Previous studies have investigated the effect of IFNα and IFNγ/IL1β on human islets using bulk RNA-seq and shown increased expression of the immunoproteasome catalytic subunits [30, 31]. In this study we found that *PSMB10* expression, responsible for INS-DRiP processing, represents a unique feature of beta cells during the late phase of type 1 diabetes, enhancing beta cell immunogenicity and discriminating beta cells that are targeted by the immune system from those that are protected. This observation adds to the accumulating evidence that the islet microenvironment acting in concert with beta cells controls immunoreactivity by altering antigen presentation pathways [26, 32]. The composition of the proteasome complex in beta cells has been studied in primary human islets and rodent insulin-producing cell lines after cytokine stimulation [33, 34]. However, few studies have connected an altered catalytic core composition with functional differences [35]. To our knowledge, our data are the first showing direct evidence for the participation of the immunoproteasome in beta cell immunogenicity and INS-DRiP-derived peptide presentation to CTLs.

Potential limitations of this study include the use of recombinant cytokines that simplify and may only partially reflect the local inflammation seen during early and late events of type 1 diabetes progression [36]. In addition, the use of a transformed human beta cell line and the limited number of autoreactive T cell clones tested mean that further validation in primary human islets in combination with other DRiP-specific T cells, when available, is required to draw broader conclusions about β2i participation in the processing of peptides derived from defective ribosomal products (currently the MLYQHLLPL peptide, studied here, remains the only alternative translational product-derived peptide identified in beta cells). Of note, while HLA-A2 expression in the EndoC-βH1 transductants [5] may not mirror normal physiological behaviour, its stable expression under different conditions allows an unbiased comparison of INS-DRiP peptide processing after IFNα or IFNɣ treatment. In addition, the selective upregulation of *PSMB10* in a subset of vulnerable beta cells reinforces the relevance of our findings and points to a role of β2i in the processing and presentation of islet neoantigens to diabetogenic T cells in type 1 diabetes.

Inhibitors targeting both the standard proteasome and the immunoproteasome, such as bortezomib, are clinically approved for the treatment of multiple myeloma and have been shown to have some beneficial effects in autoimmunity and transplantation [37–39]. However, the lack of specificity results in severe side effects, impairing their therapeutic value [40–42]. Similarly, in our analysis we observed that *PSMB9* inhibition sensitised beta cells to CTL-mediated destruction, illustrating that general interference comes at a risk and highlighting the need for specific inhibitors rather than general proteasome suppression. Immunoproteasome inhibitors have been shown to ameliorate symptoms in preclinical models of different autoimmune diseases [43, 44], and KZR-616, a drug targeting both *PSMB8* and *PSMB9*, has been tested in a Phase II trial of systemic lupus erythematosus [45]. In each study, inhibitors were used as immunosuppressants through their capacity to target immune cells that constitutively express the immunoproteasome. Some of the outcomes observed were decreased cytokine secretion, inhibition of T and B cell activation and modified macrophage polarisation [40]. While these effects may be beneficial in type 1 diabetes, a recent study showed that the use of ONX-914, a *PSMB8* inhibitor, exacerbated beta cell apoptosis during inflammation [34]. ONX-914 was later shown to have some off-target effects on *PSMB9* [46], but whether this occurred in the inflamed human islets has not yet been determined. In fact, at least two immunoproteasome subunits should be targeted to obtain successful immunosuppression [40, 46]. The use of immunoproteasome inhibitors to alleviate non-immune tissue inflammation has not yet been explored. Our study suggests that the selective modulation of MECL1 (encoded by *PSMB10*) using selective cell-permeable inhibitors of the trypsin-like site [47] may be beneficial to reduce beta cell immunogenicity and the T cell response mounted against the INS-DRiP peptide to limit type 1 diabetes progression, shedding new light on the role of the immunoproteasome, rather than the constitutive proteasome, as an important player in beta cell immunogenicity.

### Supplementary Information

Below is the link to the electronic supplementary material.

Supplementary file1 (PDF 2542 KB)

## Data Availability

The single-cell RNA-seq dataset is available from the Gene Expression Omnibus (GEO) using the accession number GSE218316 (https://www.ncbi.nlm.nih.gov/geo/query/acc.cgi?acc=GSE218316). All data are available in the main text or the electronic supplementary material.

## Abbreviations

AAb+: Autoantibody-positive
CTL: Cytotoxic T lymphocyte
DRiP: Defective ribosomal product
EF1α: Elongation factor 1α
FC: Fold change
IMDM: Iscove's modified Dulbecco's medium
INS-DRiP: Insulin-derived defective ribosomal product
LMP2: Low-molecular mass polypeptide
MECL-1: Multicatalytic endopeptidase complex-like-1
MIP-1β: Macrophage inflammatory protein-1 beta
PBMC: Peripheral blood mononuclear cells
PPI: Preproinsulin
