# Supplementary material for: IFNɣ but not IFNα increases recognition of insulin defective ribosomal product-derived antigen to amplify islet autoimmunity
Source: Diabetologia. 2023 Aug 15;66(11):2075–86. doi: 10.1007/s00125-023-05991-8 (PMC10542729; doi:10.1007/s00125-023-05991-8)
Supplement: Supplementary file 1 — Supplementary file1 (PDF 2542 KB) [file 125_2023_5991_MOESM1_ESM.pdf]

ESM table 1

Gene name	Sequence forward	Sequence Reverse
PSMB8	5'-GCTCCTGGCTGACTTCTAGT-3'	5'-GAAGAATTCTGTGGGCTCCAGG-3'
PSMB9	5'-ACAGCCTTTTGCCATTGGTG-3'	5'-GCAATAGCGTCTGTGGTGAAG-3'
PSMB10	5'-TCCAAGACGGGGTCATTCTG-3'	5'-GCCCCACAGCAGTAGATTTTG-3'
GAPDH	5'-CCTGTTCGACAGTCAGCCG-3'	5'-CGACCAAATCCGTTGACTCC-3'

ESM table 1: Primer sequences used for qPCR

ESM table 2

		IFNα					IFNγ/IL1β				
	**Gene**	**avg_logFC**	**Untreated % cells**	**IFNa % cells**	**p_val**	**p_val_adj**	**avg_logFC**	**Untreated % cells**	**IL1b+IFNg % cells**	**p_val**	**p_val_adj**
**Alpha cells**	PSMB8	0,832371501	35	75,7	4,95E-114	1,66E-109	1,038292798	35	81,6	1,16E-155	3,88E-151
	PSMB9	0,830373356	11,5	64,2	8,58E-130	2,88E-125	1,317403067	11,5	74,5	3,74E-183	1,26E-178
	PSMB10						**0,303966713**	10,4	40,4	6,80E-53	**2,28E-48**
**Beta cells**	PSMB8	0,796489248	38,7	82,6	7,69E-73	2,58E-68	1,273779973	38,7	81,2	2,26E-88	7,57E-84
	PSMB9	0,689992232	7	67,4	1,74E-84	5,82E-80	1,537457173	7	66,1	3,04E-97	1,02E-92
	PSMB10						**0,347177856**	16,6	54,4	1,59E-43	**5,33E-39**
**Delta cells**	PSMB8	0,630908942	33	73,4	1,03E-10	3,46E-06	1,075869017	33	88,3	6,05E-24	2,03E-19
	PSMB9	0,49255052	14,8	57	4,89E-10	1,64E-05	1,300417935	14,8	85,6	7,21E-26	2,42E-21
	PSMB10										
**Duct cells**	PSMB8	0,887861128	75,5	94,4	2,60E-32	8,74E-28	0,821429184	75,5	86,3	5,14E-20	1,72E-15
	PSMB9	1,255248057	50,5	95,1	1,59E-44	5,33E-40	1,354139438	50,5	73,2	2,74E-19	9,17E-15
	PSMB10										
**Acinar cells**	PSMB8	0,721706309	0,706	0,944	0,000520403	1	0,504251432	0,706	0,719	0,191748436	1
	PSMB9	0,994146578	0,485	0,944	5,26E-06	0,176283956	1,149230129	0,485	0,469	0,355174733	1
	PSMB10	-0,267028971	0,309	0,222	0,241425418	1	0,416009345	0,309	0,406	0,192125085	1

ESM table 2: Differential gene expression of Expression of the catalytic subunits of the immunoproteasome in endocrine and exocrine cells after determined after scRNA seq after IFNα and IFNɣ/IL1β stimulation (fold change >0.2). Note, no values were given for gamma and epsilon cells due to the low number of cells analyzed

ESM table 3

Cluster	ID	Description	GeneRatio	BgRatio	pvalue	p.adjust	qvalue	geneID	Count
beta1 up	R-HSA-983170	Antigen Presentation: Folding							
beta1 up	R-HSA-1236975	Antigen processing-Cross presentation	15/209	105/10899	1,28E-09	1,81E-07	1,71E-07	5698/6890/3106/5696/3105/567/3133/3134/6891/3107/6892/5720/4360/1520/8673	15
beta1 up	R-HSA-983169	Class I MHC mediated antigen processing & presentation	23/209	381/10899	1,07E-06	0,000112589	0,0001063	5698/6890/3106/5696/3105/567/3133/3134/6891/3107/6892/5720/4360/64167/51666/57520/283807/9246/8651/9021/	23
beta1 up	R-HSA-1236977	Endosomal/Vacuolar pathway	7/209	11/10899	2,67E-10	4,50E-08	4,25E-08	3106/3105/567/3133/3134/3107/1520	7
beta1 up	R-HSA-1236974	ER-Phagosome pathway	13/209	90/10899	1,46E-08	1,77E-06	1,67E-06	5698/6890/3106/5696/3105/567/3133/3134/6891/3107/6892/5720/8673	13
beta1 up	R-HSA-909733	Interferon alpha/beta signaling	16/209	72/10899	3,01E-13	2,34E-10	2,21E-10	3106/5696/3105/3133/3134/3107/25939/3661/3659/8651/10410/3660/91543/9021/2537/6772	16
beta1 up	R-HSA-877300	Interferon gamma signaling	17/209	92/10899	1,33E-12	3,74E-10	3,53E-10	3122/3106/3115/3105/567/3123/3133/3134/3107/3383/3661/3659/8651/818/3660/9021/6772	17
beta1 up	R-HSA-913531	Interferon Signaling	24/209	200/10899	5,54E-13	2,34E-10	2,21E-10	3122/3106/5696/3115/3105/567/3123/3133/3134/3107/25939/3383/3661/348995/3659/9246/8651/10410/818/3660/91	24
beta1 up	R-HSA-6785807	Interleukin-4 and Interleukin-13 signaling	9/209	108/10899	0,000223781	0,020985678	0,01981312	4609/3934/3383/8651/3576/6347/9021/4582/6772	9
beta1 up	R-HSA-2132295	MHC class II antigen presentation	9/209	123/10899	0,000588154	0,049640216	0,0468666	3122/3115/3123/1508/163/1512/972/1781/1520	9
beta2 up	R-HSA-5619084	ABC transporter disorders	7/249	78/10899	0,001984955	0,017439247	0,01428485	5698/5696/5691/5704/5720/5721/5699	7
beta2 up	R-HSA-382556	ABC-family proteins mediated transport	8/249	103/10899	0,002435265	0,020356922	0,01667478	5698/5696/5691/5704/5720/5721/1965/5699	8
beta2 up	R-HSA-176814	Activation of APC/C and APC/C:Cdc20 mediated degradation of mitotic proteins	7/249	77/10899	0,001841929	0,016349494	0,01339221	5698/5696/5691/5704/5720/5721/5699	7
beta2 up	R-HSA-1169091	Activation of NF-kappaB in B cells	8/249	67/10899	0,000135034	0,004334429	0,00355042	5698/5696/5691/5704/5720/5721/4792/5699	8
beta2 up	R-HSA-983170	Antigen Presentation: Folding							
beta2 up	R-HSA-1236975	Antigen processing-Cross presentation	17/249	105/10899	1,99E-10	1,71E-07	1,40E-07	5698/3106/5696/3105/567/3133/3134/3107/6892/5691/5704/5720/5721/4360/5699/1535/8673	17
beta2 up	R-HSA-176409	APC/C:Cdc20 mediated degradation of mitotic proteins	7/249	76/10899	0,001707031	0,015471095	0,0126727	5698/5696/5691/5704/5720/5721/5699	7
beta2 up	R-HSA-174154	APC/C:Cdc20 mediated degradation of Securin	7/249	68/10899	0,000883216	0,009875957	0,0080896	5698/5696/5691/5704/5720/5721/5699	7
beta2 up	R-HSA-174178	APC/C:Cdh1 mediated degradation of Cdc20 and other APC/C:Cdh1 targeted proteins in late mitosis/early G1	7/249	74/10899	0,001460346	0,013817117	0,01131789	5698/5696/5691/5704/5720/5721/5699	7
beta2 up	R-HSA-174143	APC/C-mediated degradation of cell cycle proteins	7/249	88/10899	0,003936107	0,029469459	0,02413905	5698/5696/5691/5704/5720/5721/5699	7
beta2 up	R-HSA-179419	APC:Cdc20 mediated degradation of cell cycle proteins prior to satisfation of the cell cycle checkpoint	7/249	74/10899	0,001460346	0,013817117	0,01131789	5698/5696/5691/5704/5720/5721/5699	7
beta2 up	R-HSA-109581	Apoptosis	15/249	180/10899	1,56E-05	0,001221976	0,00100095	5698/79792/5696/100506658/5691/5704/5720/5721/5366/637/1829/7431/1676/5699/148022	15
beta2 up	R-HSA-4608870	Asymmetric localization of PCP proteins	7/249	64/10899	0,000611431	0,007976391	0,00653363	5698/5696/5691/5704/5720/5721/5699	7
beta2 up	R-HSA-450408	AUF1 (hnRNP D0) binds and destabilizes mRNA	7/249	56/10899	0,000266492	0,005098889	0,00417661	5698/5696/5691/5704/5720/5721/5699	7
beta2 up	R-HSA-174084	Autodegradation of Cdh1 by Cdh1:APC/C	7/249	64/10899	0,000611431	0,007976391	0,00653363	5698/5696/5691/5704/5720/5721/5699	7
beta2 up	R-HSA-349425	Autodegradation of the E3 ubiquitin ligase COP1	7/249	52/10899	0,000166128	0,004334429	0,00355042	5698/5696/5691/5704/5720/5721/5699	7
beta2 up	R-HSA-174184	Cdc20:Phospho-APC/C mediated degradation of Cyclin A	7/249	73/10899	0,001347933	0,013188303	0,01080282	5698/5696/5691/5704/5720/5721/5699	7
beta2 up	R-HSA-69017	CDK-mediated phosphorylation and removal of Cdc6	7/249	73/10899	0,001347933	0,013188303	0,01080282	5698/5696/5691/5704/5720/5721/5699	7
beta2 up	R-HSA-9711123	Cellular response to chemical stress	14/249	194/10899	0,000143702	0,004334429	0,00355042	5698/5696/5691/5704/5720/5721/644/2938/7001/2878/2950/5699/1535/6648	14
beta2 up	R-HSA-1234174	Cellular response to hypoxia	8/249	75/10899	0,000298554	0,005355311	0,00438665	5698/5696/5691/5704/5720/5721/25994/5699	8
beta2 up	R-HSA-983169	Class I MHC mediated antigen processing & presentation	24/249	381/10899	6,55E-06	0,000805067	0,00065945	5698/3106/5696/3105/567/3133/3134/3107/6892/5691/5704/5720/5721/4360/55236/57630/7334/7336/122769/5699/1	24
beta2 up	R-HSA-5607764	CLEC7A (Dectin-1) signaling	10/249	101/10899	0,000100274	0,003933783	0,00322224	5698/5696/5691/5704/5720/5721/7334/4792/5699/805	10
beta2 up	R-HSA-8949613	Cristae formation	4/249	31/10899	0,005151456	0,037272302	0,03053052	79135/9551/506/10989	4
beta2 up	R-HSA-1236978	Cross-presentation of soluble exogenous antigens (endosomes)	8/249	49/10899	1,33E-05	0,001175719	0,00096306	5698/5696/5691/5704/5720/5721/4360/5699	8
beta2 up	R-HSA-5621481	C-type lectin receptors (CLRs)	11/249	142/10899	0,000405297	0,006096807	0,00499402	5698/5696/5691/5704/5720/5721/7334/4792/5699/56667/805	11
beta2 up	R-HSA-69656	Cyclin A:Cdk2-associated events at S phase entry	8/249	85/10899	0,000700224	0,008697997	0,00712471	5698/5696/5691/5704/5720/5721/4331/5699	8
beta2 up	R-HSA-69202	Cyclin E associated events during G1/S transition	8/249	83/10899	0,000596799	0,007976391	0,00653363	5698/5696/5691/5704/5720/5721/4331/5699	8
beta2 up	R-HSA-5607761	Dectin-1 mediated noncanonical NF-kB signaling	7/249	60/10899	0,000410702	0,006096807	0,00499402	5698/5696/5691/5704/5720/5721/5699	7
beta2 up	R-HSA-5678895	Defective CFTR causes cystic fibrosis	7/249	61/10899	0,00045505	0,006529961	0,00534883	5698/5696/5691/5704/5720/5721/5699	7
beta2 up	R-HSA-4641257	Degradation of AXIN	7/249	55/10899	0,000237744	0,004873762	0,0039922	5698/5696/5691/5704/5720/5721/5699	7
beta2 up	R-HSA-195253	Degradation of beta-catenin by the destruction complex	8/249	83/10899	0,000596799	0,007976391	0,00653363	5698/5696/5691/5704/5720/5721/7091/5699	8
beta2 up	R-HSA-4641258	Degradation of DVL	7/249	57/10899	0,000297972	0,005355311	0,00438665	5698/5696/5691/5704/5720/5721/5699	7
beta2 up	R-HSA-5610780	Degradation of GLI1 by the proteasome	7/249	60/10899	0,000410702	0,006096807	0,00499402	5698/5696/5691/5704/5720/5721/5699	7
beta2 up	R-HSA-5610783	Degradation of GLI2 by the proteasome	7/249	60/10899	0,000410702	0,006096807	0,00499402	5698/5696/5691/5704/5720/5721/5699	7
beta2 up	R-HSA-3299685	Detoxification of Reactive Oxygen Species	5/249	37/10899	0,001433693	0,013817117	0,01131789	7001/2878/2950/1535/6648	5
beta2 up	R-HSA-1168372	Downstream signaling events of B Cell Receptor (BCR)	10/249	83/10899	1,82E-05	0,001304911	0,00106888	5698/5696/5691/5704/5720/5721/4792/5478/5699/805	10
beta2 up	R-HSA-202424	Downstream TCR signaling	9/249	98/10899	0,000393282	0,006096807	0,00499402	5698/5696/5691/5704/5720/5721/7334/4792/5699	9
beta2 up	R-HSA-1236977	Endosomal/Vacuolar pathway	6/249	11/10899	5,62E-08	1,61E-05	1,32E-05	3106/3105/567/3133/3134/3107	6
beta2 up	R-HSA-3928663	EPHA-mediated growth cone collapse	5/249	30/10899	0,000533801	0,007412944	0,0060721	10398/10627/103910/4628/4637	5
beta2 up	R-HSA-1236974	ER-Phagosome pathway	15/249	90/10899	1,57E-09	6,78E-07	5,55E-07	5698/3106/5696/3105/567/3133/3134/3107/6892/5691/5704/5720/5721/5699/8673	15
beta2 up	R-HSA-8854050	FBXL7 down-regulates AURKA during mitotic entry and in early mitosis	7/249	54/10899	0,000211548	0,004873762	0,0039922	5698/5696/5691/5704/5720/5721/5699	7
beta2 up	R-HSA-2454202	Fc epsilon receptor (FCERI) signaling	10/249	134/10899	0,000987018	0,010622784	0,00870135	5698/5696/5691/5704/5720/5721/7334/4792/5699/805	10
beta2 up	R-HSA-2871837	FCERI mediated NF-kB activation	9/249	82/10899	0,000100515	0,003933783	0,00322224	5698/5696/5691/5704/5720/5721/7334/4792/5699	9
beta2 up	R-HSA-5696395	Formation of Incision Complex in GG-NER	5/249	43/10899	0,002833247	0,023013448	0,0188508	2966/4331/7334/7336/142	5
beta2 up	R-HSA-69615	G1/S DNA Damage Checkpoints	7/249	68/10899	0,000883216	0,009875957	0,0080896	5698/5696/5691/5704/5720/5721/5699	7
beta2 up	R-HSA-69206	G1/S Transition	9/249	131/10899	0,00308523	0,024373953	0,01996522	5698/5696/5691/5704/5720/5721/4331/51053/5699	9
beta2 up	R-HSA-8950505	Gene and protein expression by JAK-STAT signaling after Interleukin-12 stimulation	5/249	38/10899	0,0016206	0,015003616	0,01228978	5721/302/5478/6648/3181	5
beta2 up	R-HSA-5610785	GLI3 is processed to GLI3R by the proteasome	7/249	60/10899	0,000410702	0,006096807	0,00499402	5698/5696/5691/5704/5720/5721/5699	7
beta2 up	R-HSA-70263	Gluconeogenesis	4/249	34/10899	0,007198347	0,048801389	0,03997424	226/5230/7167/2805	4
beta2 up	R-HSA-9762114	GSK3B and BTRC:CUL1-mediated-degradation of NFE2L2	7/249	52/10899	0,000166128	0,004334429	0,00355042	5698/5696/5691/5704/5720/5721/5699	7
beta2 up	R-HSA-5358346	Hedgehog ligand biogenesis	7/249	65/10899	0,000672107	0,008510062	0,00697077	5698/5696/5691/5704/5720/5721/5699	7
beta2 up	R-HSA-5610787	Hedgehog 'off' state	9/249	113/10899	0,00110985	0,01179729	0,00966341	5698/5696/5691/5704/5720/5721/51098/5699/55764	9
beta2 up	R-HSA-5632684	Hedgehog 'on' state	7/249	85/10899	0,00324165	0,025144693	0,02059655	5698/5696/5691/5704/5720/5721/5699	7
beta2 up	R-HSA-5387390	Hh mutants abrogate ligand secretion	7/249	59/10899	0,000369879	0,006096807	0,00499402	5698/5696/5691/5704/5720/5721/5699	7
beta2 up	R-HSA-5362768	Hh mutants are degraded by ERAD	7/249	56/10899	0,000266492	0,005098889	0,00417661	5698/5696/5691/5704/5720/5721/5699	7
beta2 up	R-HSA-162906	HIV Infection	12/249	231/10899	0,006792868	0,047166608	0,03863516	2966/5698/5696/3105/567/5691/5704/5720/5721/4331/5478/5699	12
beta2 up	R-HSA-162909	Host Interactions of HIV factors	10/249	130/10899	0,000780686	0,009083388	0,00744039	5698/5696/3105/567/5691/5704/5720/5721/5478/5699	10
beta2 up	R-HSA-9635486	Infection with Mycobacterium tuberculosis	4/249	27/10899	0,003085669	0,024373953	0,01996522	567/4360/5230/57674	4
beta2 up	R-HSA-216083	Integrin cell surface interactions	7/249	85/10899	0,00324165	0,025144693	0,02059655	3696/3383/3688/682/6696/960/961	7
beta2 up	R-HSA-909733	Interferon alpha/beta signaling	10/249	72/10899	5,00E-06	0,000734621	0,00060174	3106/5696/3105/3133/3134/3107/10410/91543/2537/9636	10
beta2 up	R-HSA-877300	Interferon gamma signaling	9/249	92/10899	0,000244455	0,004894795	0,00400943	3106/3105/567/3133/3134/3107/3383/818/960	9
beta2 up	R-HSA-913531	Interferon Signaling	15/249	200/10899	5,37E-05	0,002891085	0,00236815	3106/5696/3105/567/3133/3134/3107/3383/7334/10410/818/91543/960/2537/9636	15
beta2 up	R-HSA-446652	Interleukin-1 family signaling	10/249	153/10899	0,002665025	0,021853202	0,01790042	5698/79792/5696/5691/5704/5720/5721/7334/4792/5699	10
beta2 up	R-HSA-9020702	Interleukin-1 signaling	9/249	115/10899	0,001256805	0,012730699	0,01042798	5698/5696/5691/5704/5720/5721/7334/4792/5699	9
beta2 up	R-HSA-9020591	Interleukin-12 signaling	5/249	47/10899	0,004193567	0,030860353	0,02527836	5721/302/5478/6648/3181	5
beta2 up	R-HSA-9755511	KEAP1-NFE2L2 pathway	8/249	102/10899	0,002290254	0,019523845	0,01599239	5698/5696/5691/5704/5720/5721/2938/5699	8
beta2 up	R-HSA-5687128	MAPK6/MAPK4 signaling	7/249	89/10899	0,004190988	0,030860353	0,02527836	5698/5696/5691/5704/5720/5721/5699	7
beta2 up	R-HSA-351202	Metabolism of polyamines	8/249	59/10899	5,37E-05	0,002891085	0,00236815	5698/5696/5691/5704/5720/5721/6303/5699	8
beta2 up	R-HSA-1592230	Mitochondrial biogenesis	7/249	95/10899	0,005991688	0,042635067	0,03492328	79135/9551/3418/506/6648/10989/805	7
beta2 up	R-HSA-5368287	Mitochondrial translation	7/249	93/10899	0,005337277	0,038294963	0,03136821	28998/92399/10573/51264/26284/79590/64432	7
beta2 up	R-HSA-5419276	Mitochondrial translation termination	7/249	87/10899	0,003693166	0,028139961	0,02305003	28998/92399/10573/51264/26284/79590/64432	7
beta2 up	R-HSA-453279	Mitotic G1 phase and G1/S transition	9/249	149/10899	0,007153859	0,048801389	0,03997424	5698/5696/5691/5704/5720/5721/4331/51053/5699	9
beta2 up	R-HSA-9604323	Negative regulation of NOTCH4 signaling	7/249	54/10899	0,000211548	0,004873762	0,0039922	5698/5696/5691/5704/5720/5721/5699	7
beta2 up	R-HSA-6798695	Neutrophil degranulation	28/249	482/10899	5,12E-06	0,000734621	0,00060174	3106/79792/3105/567/3107/7276/2171/2720/5476/4257/51071/2519/58485/84418/2717/226/302/374395/5478/9961/2	28
beta2 up	R-HSA-5676590	NIK-->noncanonical NF-kB signaling	7/249	59/10899	0,000369879	0,006096807	0,00499402	5698/5696/5691/5704/5720/5721/5699	7
beta2 up	R-HSA-9759194	Nuclear events mediated by NFE2L2	8/249	79/10899	0,000426879	0,006229546	0,00510275	5698/5696/5691/5704/5720/5721/2938/5699	8
beta2 up	R-HSA-68949	Orc1 removal from chromatin	7/249	71/10899	0,001143452	0,012006249	0,00983457	5698/5696/5691/5704/5720/5721/5699	7
beta2 up	R-HSA-1234176	Oxygen-dependent proline hydroxylation of Hypoxia-inducible Factor Alpha	7/249	66/10899	0,000737461	0,008697997	0,00712471	5698/5696/5691/5704/5720/5721/5699	7
beta2 up	R-HSA-69563	p53-Dependent G1 DNA Damage Response	7/249	66/10899	0,000737461	0,008697997	0,00712471	5698/5696/5691/5704/5720/5721/5699	7
beta2 up	R-HSA-69580	p53-Dependent G1/S DNA damage checkpoint	7/249	66/10899	0,000737461	0,008697997	0,00712471	5698/5696/5691/5704/5720/5721/5699	7
beta2 up	R-HSA-69610	p53-Independent DNA Damage Response	7/249	52/10899	0,000166128	0,004334429	0,00355042	5698/5696/5691/5704/5720/5721/5699	7
beta2 up	R-HSA-69613	p53-Independent G1/S DNA damage checkpoint	7/249	52/10899	0,000166128	0,004334429	0,00355042	5698/5696/5691/5704/5720/5721/5699	7
beta2 up	R-HSA-4086400	PCP/CE pathway	7/249	92/10899	0,005030949	0,03670887	0,03006901	5698/5696/5691/5704/5720/5721/5699	7
beta2 up	R-HSA-114608	Platelet degranulation	10/249	129/10899	0,000735086	0,008697997	0,00712471	7076/350/226/7114/1191/710/5478/3959/805/5768	10
beta2 up	R-HSA-5357801	Programmed Cell Death	16/249	210/10899	2,49E-05	0,00165167	0,00135292	5698/79792/5696/100506658/5691/5704/5720/5721/5366/637/1829/7431/1676/837/5699/148022	16
beta2 up	R-HSA-71406	Pyruvate metabolism and Citric Acid (TCA) cycle	6/249	55/10899	0,001520637	0,01423118	0,01165706	3939/50/6392/682/3418/5166	6
beta2 up	R-HSA-211733	Regulation of activated PAK-2p34 by proteasome mediated degradation	7/249	50/10899	0,000128941	0,004334429	0,00355042	5698/5696/5691/5704/5720/5721/5699	7
beta2 up	R-HSA-176408	Regulation of APC/C activators between G1/S and early anaphase	7/249	81/10899	0,00246608	0,020416299	0,01672342	5698/5696/5691/5704/5720/5721/5699	7
beta2 up	R-HSA-169911	Regulation of Apoptosis	7/249	53/10899	0,000187731	0,004754008	0,00389411	5698/5696/5691/5704/5720/5721/5699	7
beta2 up	R-HSA-453276	Regulation of mitotic cell cycle	7/249	88/10899	0,003936107	0,029469459	0,02413905	5698/5696/5691/5704/5720/5721/5699	7
beta2 up	R-HSA-450531	Regulation of mRNA stability by proteins that bind AU-rich elements	10/249	88/10899	3,05E-05	0,001878156	0,00153844	5698/5696/5691/5704/5720/5721/11340/7538/677/5699	10
beta2 up	R-HSA-350562	Regulation of ornithine decarboxylase (ODC)	7/249	51/10899	0,000146582	0,004334429	0,00355042	5698/5696/5691/5704/5720/5721/5699	7
beta2 up	R-HSA-8948751	Regulation of PTEN stability and activity	7/249	69/10899	0,000964142	0,010622784	0,00870135	5698/5696/5691/5704/5720/5721/5699	7
beta2 up	R-HSA-5658442	Regulation of RAS by GAPs	7/249	68/10899	0,000883216	0,009875957	0,0080896	5698/5696/5691/5704/5720/5721/5699	7
beta2 up	R-HSA-8939902	Regulation of RUNX2 expression and activity	7/249	73/10899	0,001347933	0,013188303	0,01080282	5698/5696/5691/5704/5720/5721/5699	7
beta2 up	R-HSA-8941858	Regulation of RUNX3 expression and activity	7/249	55/10899	0,000237744	0,004873762	0,0039922	5698/5696/5691/5704/5720/5721/5699	7
beta2 up	R-HSA-163200	Respiratory electron transport							
beta2 up	R-HSA-611105	Respiratory electron transport	8/249	103/10899	0,002435265	0,020356922	0,01667478	4696/29078/4694/7385/6392/51103/2108/4704	8
beta2 up	R-HSA-76005	Response to elevated platelet cytosolic Ca2+	10/249	134/10899	0,000987018	0,010622784	0,00870135	7076/350/226/7114/1191/710/5478/3959/805/5768	10
beta2 up	R-HSA-5625900	RHO GTPases activate CIT	5/249	20/10899	7,00E-05	0,00334782	0,00274227	10398/10627/103910/4628/4637	5
beta2 up	R-HSA-5627123	RHO GTPases activate PAKs	6/249	24/10899	1,28E-05	0,001175719	0,00096306	10398/10627/103910/4628/4637/805	6
beta2 up	R-HSA-5627117	RHO GTPases Activate ROCKs	5/249	20/10899	7,00E-05	0,00334782	0,00274227	10398/10627/103910/4628/4637	5
beta2 up	R-HSA-187577	SCF(Skp2)-mediated degradation of p27/p21	7/249	60/10899	0,000410702	0,006096807	0,00499402	5698/5696/5691/5704/5720/5721/5699	7

beta2	up	R-HSA-174113	SCF-beta-TrCP mediated degradation of Emi1	7/249	55/10899	0,000237744	0,004873762	0,0039922	5698/5696/5691/5704/5720/5721/5699	7
beta2	up	R-HSA-400685	Sema4D in semaphorin signaling	5/249	25/10899	0,000218451	0,004873762	0,0039922	10398/10627/103910/4628/4637	5
beta2	up	R-HSA-416572	Sema4D induced cell migration and growth-cone collapse	5/249	21/10899	9,02E-05	0,003933783	0,00322224	10398/10627/103910/4628/4637	5
beta2	up	R-HSA-373755	Semaphorin interactions	6/249	65/10899	0,003585862	0,027566316	0,02258015	10398/10627/103910/3688/4628/4637	6
beta2	up	R-HSA-5358351	Signaling by Hedgehog	9/249	149/10899	0,007153859	0,048801389	0,03997424	5698/5696/5691/5704/5720/5721/51098/5699/55764	9
beta2	up	R-HSA-449147	Signaling by Interleukins	22/249	473/10899	0,001223187	0,012537672	0,01026987	5698/79792/5696/5691/5704/5720/5721/4692/7076/3383/7334/3688/302/7431/4792/2920/9235/5478/3576/5699/6648	22
beta2	up	R-HSA-157118	Signaling by NOTCH	13/249	236/10899	0,003001212	0,024149942	0,01978172	5698/5696/5691/5704/5720/5721/3280/92737/55851/7091/5699/4192/1999	13
beta2	up	R-HSA-9013694	Signaling by NOTCH4	9/249	82/10899	0,000100515	0,003933783	0,00322224	5698/5696/5691/5704/5720/5721/3280/55851/5699	9
beta2	up	R-HSA-983705	Signaling by the B Cell Receptor (BCR)	10/249	112/10899	0,000237225	0,004873762	0,0039922	5698/5696/5691/5704/5720/5721/4792/5478/5699/805	10
beta2	up	R-HSA-445355	Smooth Muscle Contraction	6/249	45/10899	0,000516639	0,007292235	0,00597322	10398/10627/103910/302/4637/805	6
beta2	up	R-HSA-69541	Stabilization of p53	7/249	57/10899	0,000297972	0,005355311	0,00438665	5698/5696/5691/5704/5720/5721/5699	7
beta2	up	R-HSA-69052	Switching of origins to a post-replicative state	8/249	92/10899	0,001181278	0,012253979	0,01003749	5698/5696/5691/5704/5720/5721/51053/5699	8
beta2	up	R-HSA-69239	Synthesis of DNA	8/249	121/10899	0,006498605	0,045490237	0,03726201	5698/5696/5691/5704/5720/5721/51053/5699	8
beta2	up	R-HSA-202403	TCR signaling	9/249	120/10899	0,001693736	0,015471095	0,0126727	5698/5696/5691/5704/5720/5721/7334/4792/5699	9
beta2	up	R-HSA-1428517	The citric acid (TCA) cycle and respiratory electron transport	15/249	178/10899	1,37E-05	0,001175719	0,00096306	4696/3939/29078/50/4694/7385/6392/51103/682/9551/3418/506/2108/5166/4704	15
beta2	up	R-HSA-8852276	The role of GTSE1 in G2/M progression after G2 checkpoint	7/249	77/10899	0,001841929	0,016349494	0,01339221	5698/5696/5691/5704/5720/5721/5699	7
beta2	up	R-HSA-5668541	TNFR2 non-canonical NF-kB pathway	8/249	102/10899	0,002290254	0,019523845	0,01599239	5698/5696/5691/5704/5720/5721/51330/5699	8
beta2	up	R-HSA-8878166	Transcriptional regulation by RUNX2	8/249	121/10899	0,006498605	0,045490237	0,03726201	5698/5696/5691/5704/5720/5721/3280/5699	8
beta2	up	R-HSA-8878159	Transcriptional regulation by RUNX3	9/249	96/10899	0,000337087	0,005923103	0,00485174	5698/5696/5691/5704/5720/5721/3280/6696/5699	9
beta2	up	R-HSA-69601	Ubiquitin Mediated Degradation of Phosphorylated Cdc25A	7/249	52/10899	0,000166128	0,004334429	0,00355042	5698/5696/5691/5704/5720/5721/5699	7
beta2	up	R-HSA-75815	Ubiquitin-dependent degradation of Cyclin D	7/249	52/10899	0,000166128	0,004334429	0,00355042	5698/5696/5691/5704/5720/5721/5699	7
beta2	up	R-HSA-5689603	UCH proteinases	8/249	102/10899	0,002290254	0,019523845	0,01599239	5698/5696/5691/5704/5720/5721/8607/5699	8
beta2	up	R-HSA-180585	Vif-mediated degradation of APOBEC3G	7/249	54/10899	0,000211548	0,004873762	0,0039922	5698/5696/5691/5704/5720/5721/5699	7
beta2	up	R-HSA-180534	Vpu mediated degradation of CD4	7/249	52/10899	0,000166128	0,004334429	0,00355042	5698/5696/5691/5704/5720/5721/5699	7

ESM table 3: Reactome analysis of the upregulated genes in beta1 and beta2 subsets identified by Fasolino et al. 2022 (pvalue=0,05; logFC>1), generated using R package Clusterprofiler and enrichPathway function. Common pathways upregulated in beta1 and beta2 are highlighted in yellow. Specific pathways upregulated in beta1 are highlighted in green.

ESM figure 1.

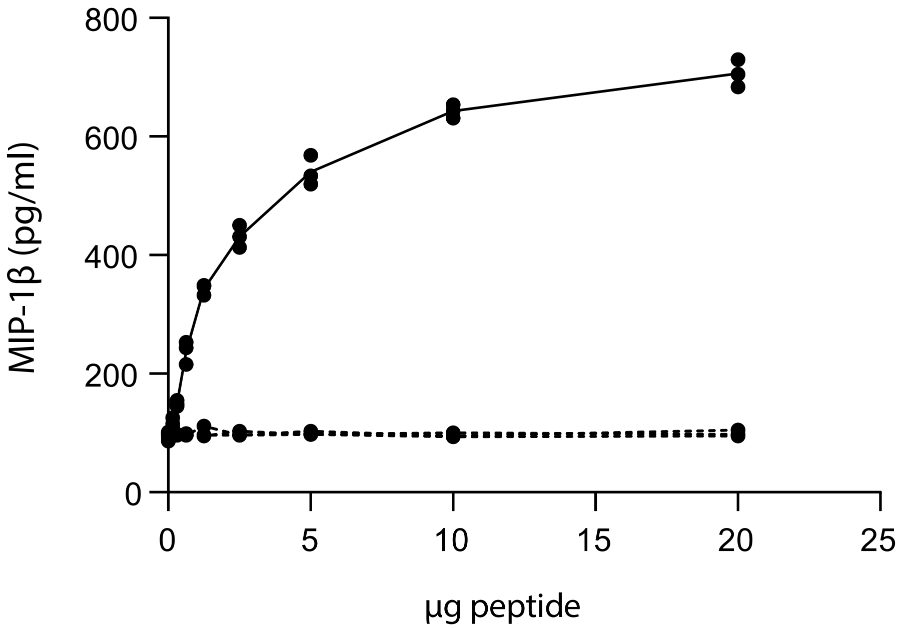

ESM figure 1. Peptide surface concentration affects INS-DRIP CTL activation. MIP-1β secretion of INS-DRiP–specific CTLs after coculture with JY-HLA-A2 pulsed with increasing concentrations of INS-DRIP1-9 (black line) or PPI15-24 (dashed line, control) peptide.

ESM figure 2.

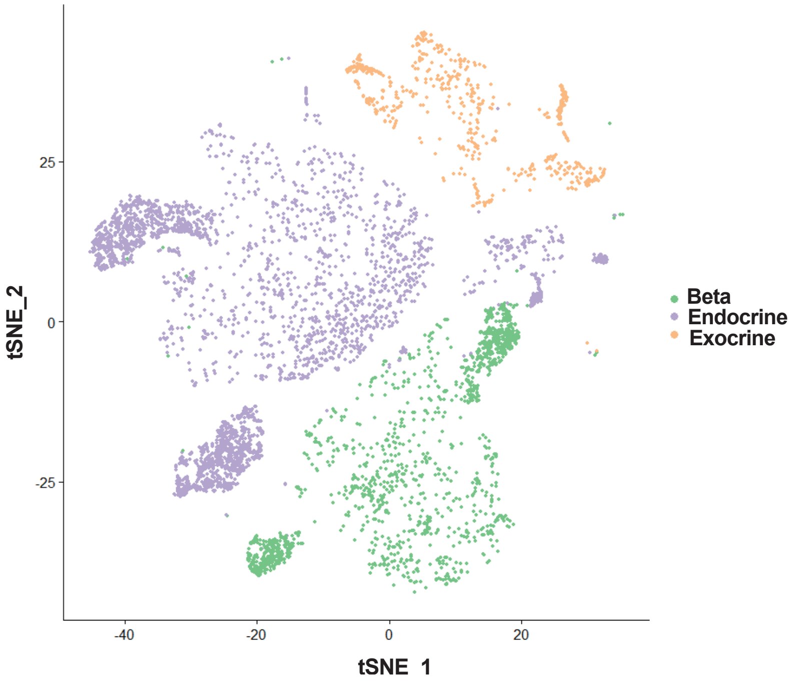

ESM figure 2. Clustering of single cell RNA-sequencing. Representative tSNE plot of cell population clusters as found in single cell RNA-sequencing. Cell color specifies assignment of the cells into one of the three clusters: beta-cells, endocrine (alpha, delta, and gamma cells) and exocrine (duct and acinar cells).

ESM figure 3.

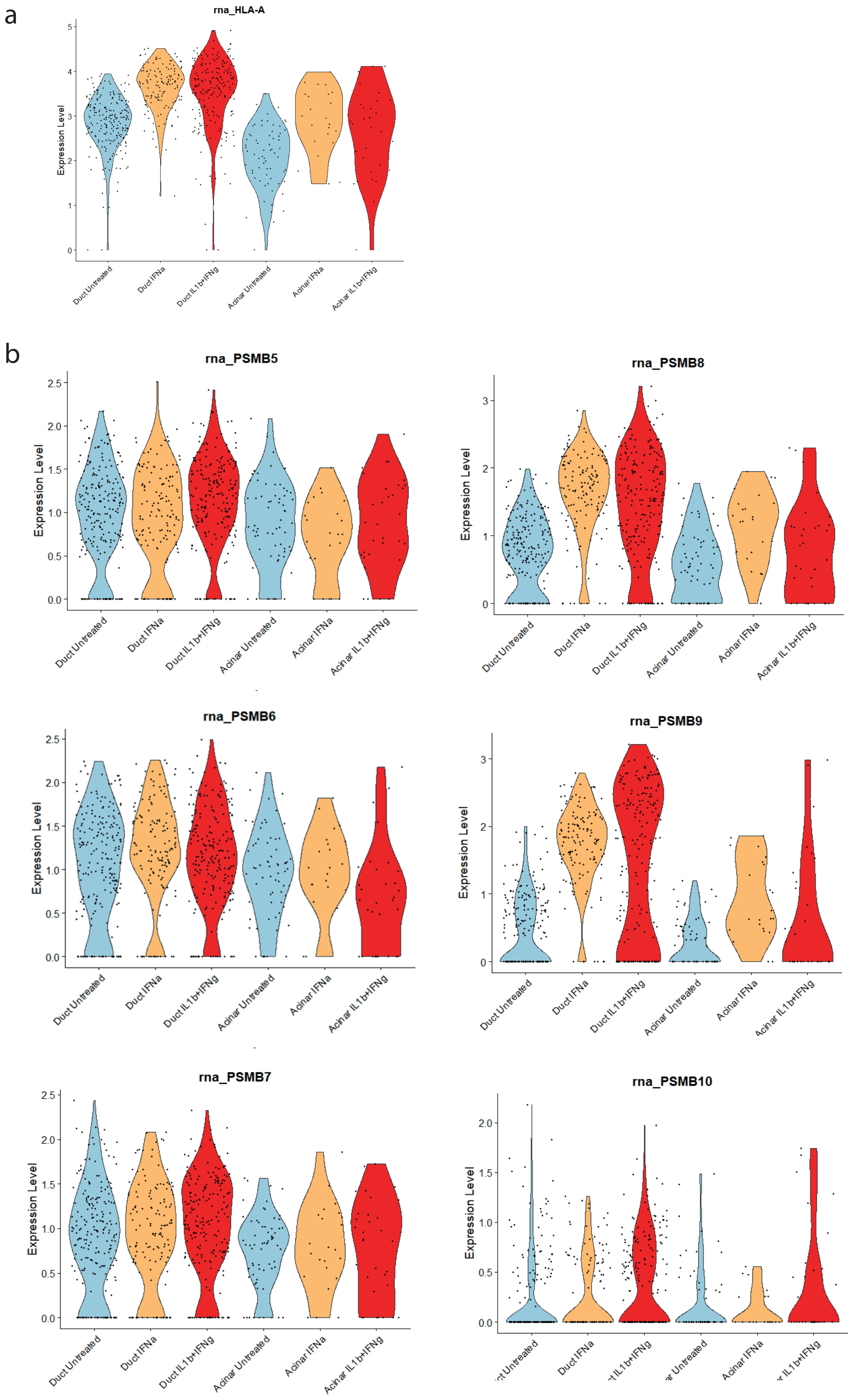

ESM figure 3. Expression of the immunoproteasome catalytic subunits expression in primary exocrine cells. (a) Violin plots showing the expression levels of *HLA- A* after IFNα or IFNγ/IL1β treatment in exocrine cells. (b) Violin plots showing the expression levels of the constitutive (*PSMB5,6* and *7*) and induced (*PSMB8, 9* and *10*) catalytic subunits of the proteasome. Expression levels correspond to logUMI counts/cell, as obtained in single cell RNA sequencing of human islets treated with IFNα or IFNγ+IL1β for 24 hours.

ESM figure 4.

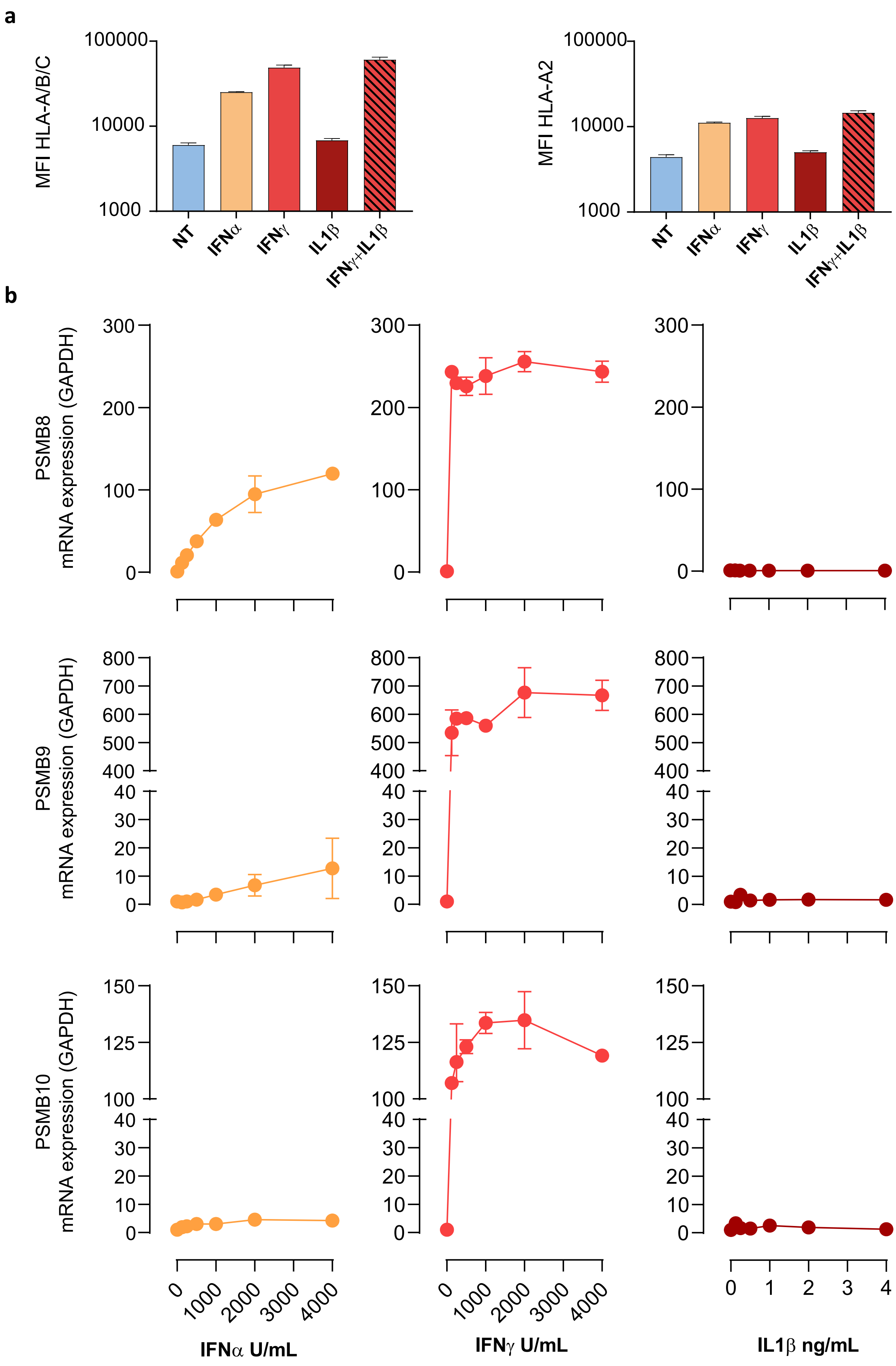

ESM figure 4: Effect of proinflammatory cytokines on HLA molecules and catalytic subunits of the immunoproteasome. a) HLA A/B/C and HLA-A2 surface expression in EndoC-BH1/A2 cells after exposure to IFNα (2000 U/ml), IFNγ (1000 U/ml) and IL1β (1 ng/ml) for 24 hours. b) Expression of the immunoproteasome catalytic subunits in EndoC-β H1 cells, determined by qPCR, after exposure to increasing amount of IFNα (125; 250; 500; 1000; 2000; 4000 U/mL), IFNγ (125; 250; 500; 1000; 2000; 4000 U/mL) and IL1β (0,125; 0,25; 0,5; 1; 2, 4ng/mL) for 24h. GAPDH was used as reference.

ESM figure 5.

a

>INS_DRIP				
Aminoacid	Proteasome Cleavage	Score	Immunoproteasome cleavage	Score
M	YES	0.5697	NO	0.3644
L	YES	0.6722	YES	0.6597
Y	YES	0.8172	YES	0.6272
Q	YES	0.5008	NO	0.0882
H	NO	0.0017	NO	0.0189
L	YES	0.5278	YES	0.5577
L	NO	0.1653	YES	0.8052
P	NO	0.0209	NO	0.0355
L	NO	0.1653	YES	0.8052
P	NO	0.0292	NO	0.0391
A	NO	0.3388	NO	0.2849
G	NO	0.0333	NO	0.0327
E	NO	0.0138	NO	0.0117
L	YES	0.5278	YES	0.5577
L	YES	0.5899	YES	0.7165
Q	NO	0.0137	NO	0.0492
L	YES	0.5843	YES	0.6509
D	NO	0.0489	NO	0.0282
A	NO	0.2652	NO	0.2540
A	NO	0.2950	NO	0.3659
R	YES	0.5294	NO	0.1274
R	YES	0.5127	NO	0.1963
Q	NO	0.0021	NO	0.0805
P	NO	0.0283	NO	0.0596
H	NO	0.0023	NO	0.0620
T	NO	0.0382	NO	0.1001
R	YES	0.5294	NO	0.1274
R	NO	0.4214	NO	0.1878
L	YES	0.5278	YES	0.5577
L	YES	0.6459	YES	0.6499
H	NO	0.0042	NO	0.0224
R	YES	0.5086	NO	0.2924
E	NO	0.0256	NO	0.1274
R	NO	0.3822	YES	0.5309
W	YES	0.5889	YES	0.8442
N	NO	0.0351	NO	0.0186
K	NO	0.3776	YES	0.6640
A	NO	0.1134	NO	0.2278
L	YES	0.5983	YES	0.6883
E	NO	0.0019	NO	0.0198
P	NO	0.0248	NO	0.0296

b

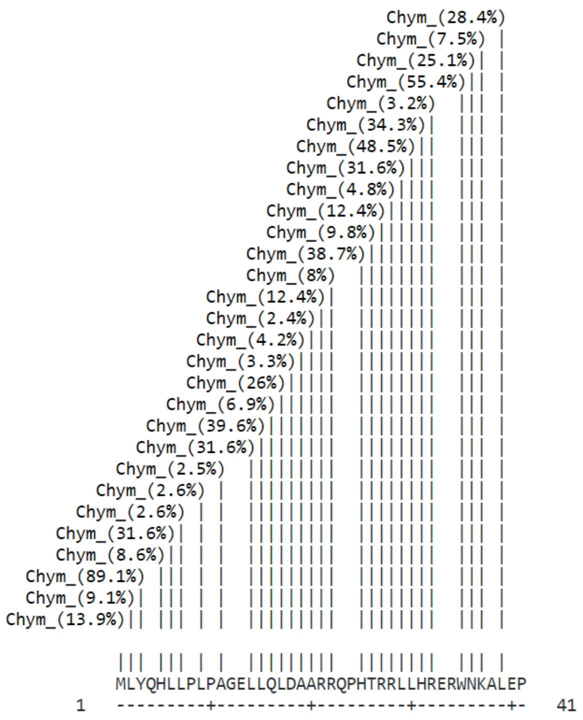

C.

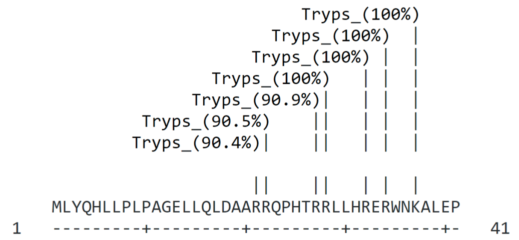

ESM figure 5. In silico prediction of INS-DRiP processing. (a) Proteasome and immunoproteasome cleavage prediction from the PCPS server. “YES”: potential cleavage site, “NO”: no cleavage, Score: Cleavage score of the C-terminal side of the residue, positive threshold > 0.5. (b) Chymotrypsin (putative β5i, β1i activity) cleavage site prediction from the ExPASy peptide cutter platform. (c) Trypsin (putative β2i activity) cleavage site prediction from the ExPASy peptide cutter platform. Percentages indicate the cleavage probability on the C-terminal side of the residue.

Diabetologia

Checklist for reporting human islet preparations used in research

Adapted from Hart NJ, Powers AC (2018) Progress, challenges, and suggestions for using human islets to understand islet biology and human diabetes. Diabetologia https://doi.org/10.1007/s00125-018-4772-2

**Islet preparation**	**1**	**2**	**3**	**4**	**5**	**6**	**7**	**8[a]**
**MANDATORY INFORMATION**								
Unique identifier								
Donor age (years)	31							
Donor sex (M/F)	F							
Donor BMI (kg/m^2)	26							
Donor HbA_{1c} or other measure of blood glucose control	10mM (latest measurement at ICU)							
Origin/source of islets[b]								
Islet isolation centre	LUMC							
Donor history of diabetes? Please select yes/no from drop down list	No							
**If Yes, complete the next two lines if this information is available**								
Diabetes duration (years)								
Glucose-lowering therapy at time of death[c]								
**RECOMMENDED INFORMATION**								
Donor cause of death								
Warm ischaemia time (h)								
Cold ischaemia time (h)								

Estimated purity (%)	75%							
Estimated viability (%)								
Total culture time (h)[d]								
Glucose-stimulated insulin secretion or other functional measurement[e]								
Handpicked to purity? Please select yes/no from drop down list								
Additional notes								

[a]If you have used more than eight islet preparations, please complete additional forms as necessary
[b]For example, IIDP, ECIT, Alberta IsletCore
[c]Please specify the therapy/therapies
[d]Time of islet culture at the isolation centre, during shipment and at the receiving laboratory
[e]Please specify the test and the results
